# Open-Circuit Fault Diagnosis of T-Type Three-Level Inverter Based on Knowledge Reduction

**DOI:** 10.3390/s24031028

**Published:** 2024-02-05

**Authors:** Xiaojuan Chen, Zhaohua Zhang

**Affiliations:** School of Electronic Information Engineering, Changchun University of Science and Technology, Changchun 130022, China; 2022200110@mails.cust.edu.cn

**Keywords:** inverter, fault diagnosis, knowledge reduction, open circuit

## Abstract

Compared with traditional two-level inverters, multilevel inverters have many solid-state switches and complex composition methods. Therefore, diagnosing and treating inverter faults is a prerequisite for the reliable and efficient operation of the inverter. Based on the idea of intelligent complementary fusion, this paper combines the genetic algorithm–binary granulation matrix knowledge-reduction method with the extreme learning machine network to propose a fault-diagnosis method for multi-tube open-circuit faults in T-type three-level inverters. First, the fault characteristics of power devices at different locations of T-type three-level inverters are analyzed, and the inverter output power and its harmonic components are extracted as the basis for power device fault diagnosis. Second, the genetic algorithm–binary granularity matrix knowledge-reduction method is used for optimization to obtain the minimum attribute set required to distinguish the state transitions in various fault cases. Finally, the kernel attribute set is utilized to construct extreme learning machine subclassifiers with corresponding granularity. The experimental results show that the classification accuracy after attribute reduction is higher than that of all subclassifiers under different attribute sets, reflecting the advantages of attribute reduction and the complementarity of different intelligent diagnosis methods, which have stronger fault-diagnosis accuracy and generalization ability compared with the existing methods and provides a new way for hybrid intelligent diagnosis.

## 1. Introduction

The multilevel inverter is a power electronic device that generates output voltage waveforms and current waveforms using a variety of direct current (DC) voltage sources and power switches [1]. Multilevel inverters are widely used in low-voltage situations and mid-frequency switching frequency scenarios due to their advantages of low switching transient voltage change rate, small harmonic distortion, and high power conversion efficiency [2], and have become indispensable electronic devices in power systems as modern industry develops in the direction of scale, accuracy, systematization, automation, and intelligence. The complexity of the T-type topology and the abundance of power semiconductor devices have recently raised questions about the ability of the system to operate reliably in comparison to the traditional two-level inverter [3].

Because semiconductor power devices are relatively fragile, open-circuit (OC) faults and short-circuit (SC) faults of insulated gate bipolar transistors (IGBTs) can be distinguished based on their external behavior [4]. SC faults cause short circuits and abnormal overcurrent states, causing other components to be damaged. It is necessary to isolate the problematic component or to immediately shut down the whole system, for instance, using desaturation detection in the door driver or fast fuses [5]. On the contrary, OC fault may not immediately cause system failure but may cause current distortion and secondary damage to other components due to increased noise and voltage stress. As a result, effective OC fault diagnosis is critical for improving power system reliability.

At present, more methods based on signal processing are being developed to decompose, convert, or lower the dimension of system detection data according to the signal analysis strategy and extract feature information from it. Fault diagnosis and identification are achieved by comparing the changing pattern of feature information before and after the fault. The authors in [6] proposed a non-invasive diagnostic strategy to detect the near-field voltage signal of the inverter DC bus through the antenna and extract the spectral features of the collected signal using fast Fourier transform (FFT) as a basis for fault classification. However, due to the limitation of the amount of diagnostic information, this method can only achieve the diagnosis of the clamping diode open-circuit fault. A discrete wavelet transform-based fault feature extraction strategy for microgrid inverters was proposed by the authors in [7]. The authors in [8] propose a fault-diagnosis method based on the average modulation voltage model for multi-current sensor disordered grid-connected inverters, which establishes the average modulation voltage model of three-phase steady-state coordinates and estimates the difference between the measured value and the actual value of the current by the model. Then, the fault is found. The experimental results show that the method can accurately locate the fault and perform fault-tolerant control when multiple sensors have offset faults at the same time. By symmetrically reconstructing the phase current signal, the effect of load variation is eliminated while retaining the main features of the fault. Then, multi-scale feature extraction is performed on the signal, and the energy coefficients of each group of current signals at different frequencies obtained are used as diagnostic classification information. However, the selection of wavelet bases will directly affect the extraction effect of fault features, which increases the difficulty of applying this method. To accurately detect IGBT switching faults, a new method based on an enhanced version of the variable mode decomposition algorithm (EVMD) combined with wavelet packet analysis (WPA) and scalar indicators is proposed by the authors in [9] to detect OC faults, which also shows how effective the suggested method is at diagnosing OC faults. For three-level active neutral-point-clamped (3LANPC) inverters, the authors in [10] established a predictive current model and seamlessly integrated the residuals of the predicted current vectors between the measured and predicted currents into the backward optimization of the MPC to diagnose inverter faults, which reduces the complexity of inverter fault identification, while the authors defined the counting function within each current cycle, which enhances the robustness of the algorithm. However, the method proposed in the article was based on generalized current residuals and a fault hypothesis prediction model. In [11], a method based on an average voltage vector was proposed, in which the threshold value is established by vector trajectory prediction, and the diagnostic variables include neutral point potential, eigenvector angle, and eigenvector modulus. These methods, however, all rely on signals provided by the system controller, resulting in a lengthy diagnosis time. To address this issue, the authors in [12] proposed using simple logic circuits to process the voltage and switching signals of the upper bridge transistors, as well as adding hardware to the inverter to provide transient fault information, but this introduced additional costs and complexity. Following that, a model simulation that infers system operation is proposed. For example, consider the hybrid logic dynamic diagnosis model, which is made up of a two-level inverter and an NPC inverter [13,14]. The diagnostic signal is defined as the difference between the sampling and estimated currents, and the fault location is determined by the residual change rate. In [15], branch level and equipment level faults are identified hierarchically using the DC-link model, and parameter errors like inductance error and sampling error are processed to ensure accurate diagnosis results while also enhancing diagnosis speed and robustness. However, OC faults in different inverter transistors can produce similar fault characteristics [16].

Some artificial intelligence methods are used for state feature classification and are becoming a prominent research area as machine-learning (ML) technology and computing capacity grow. The authors in [17] made improvements to convolutional neural networks using a global average pooling layer instead of a fully connected layer, and the improved method reduces the number of model parameters of traditional neural networks greatly, which is beneficial to achieving fast fault diagnosis of inverters. To further optimize the diagnostic performance and improve diagnostic accuracy, its integrated processing and collaborative analysis are used for inverter fault diagnosis, which is a common information fusion process. The combination of different algorithms is used to enhance the extraction of fault feature information and to improve the classification and discrimination of fault features at the same time. The authors in [18] carried out data processing and model construction for inverter open-circuit faults. In order to increase the number of samples, the authors used a Conditional Variational Auto-Encoder for data enhancement of the fault samples and Wavelet Packet Decomposition to eliminate the noise in the samples; then, the authors constructed an improved residual network with a channel attention module as a fault-diagnosis model, and the simulation results show that targeting the inverter has higher diagnostic accuracy, faster convergence speed and shorter iteration period in fault diagnosis. However, the methodology used by the authors is more stringent on the accuracy of the dataset. Any deviation or error present in these initial fault datasets may affect the accuracy of the final fault diagnosis. The authors in [19] proposed a neural network diagnosis strategy based on a circuit resolution model. This method combines the advantages of both circuit analysis and data-driven diagnostic strategies, derives diagnostic signals that directly reflect circuit fault patterns through the parsing model, and then uses Artificial Neural Network (ANN) to identify and classify the feature information in the diagnostic signals, avoiding complex fault analysis, rule specification, and threshold selection problems. Abdo, Ali, and colleagues proposed improving fault classification accuracy by optimizing the data itself [20]. In [21], a long short-term memory (LSTM) neural network and a clustering algorithm were used to create a neural network model for fault detection. To locate faults, the authors in [22] combined a deep convolutional network with network topology. Zhou et al. then used a granular Markov model to detect anomalous behavior after being inspired by the thought of information granularity [23]. For neutral-point-clamped inverters, the authors in [24] proposed a data-driven inverter fault-diagnosis method based on the design of labels to simplify the traditional labeling method and one-dimensional depth-separable convolution (1D-DSC) and global maximum pooling (GMP) methods to process the data. Then, the TensorRT framework is used for model compression and optimization. Simulation results show that the proposed method can reduce the number of model parameters by more than 90% and has better online application potential for fault diagnosis. The authors in [25] combined two methods of chaotic adaptive gravity search algorithm (GSA) and back propagation neural network (BPNN) optimized by particle swarm optimization(PSO) algorithm to establish a fault-diagnosis model based on chaotic adaptive GSA-PSO-BPNN, which improved the fault classification performance, and the feasibility and effectiveness of the algorithm was demonstrated. Although the above literature does not require the analysis of the circuit operation mechanism or the creation of an accurate circuit model, the diagnosis time for faults is generally long. This is because complex calculations are generally performed on many diagnostic signals to accurately identify fault characteristics, a process that requires long data acquisition time and signal processing time.

Power inverters, on the other hand, are more complex systems, making it challenging to collect complete experimental data for fault diagnosis. Rough set theory is a new mathematical tool that can be used to deal with fuzzy and uncertain knowledge and has strong qualitative analysis capabilities. Rough sets can be directly analyzed and reasoned from experimental data to discover a large amount of implicit information knowledge and reveal the inherent law. Rough set attribute reduction has been used to help diagnose power transformer faults in recent years, with some success [26,27].

To summarize, artificial intelligence-based approaches may learn the nonlinear relationship between faults and fault features from data and have better diagnostic detection capabilities. However, when the neural network method is applied in the field of fault diagnosis, training samples are not easy to obtain, and it is difficult to perfectly integrate all expert experience and knowledge, making the diagnosis inaccurate and low precision. Integrated artificial intelligence and signal processing combine the advantages of different strategies. The required data are fewer, and the diagnostic model structure is relatively simple, but it still takes more than half the fundamental wave period to locate the fault. This speed is difficult to meet on some occasions that require high real-time performance of fault protection isolation or fault tolerance. In contrast, the most important feature of the rough set method is that it can objectively describe and process uncertain events without requiring subjective a priori information outside the data-set, and the core element of the method is attribute parsimony. In inverter fault diagnosis, rough set attribute simplification is usually used to reduce the dimensionality of feature quantities and reduce the size and complexity of the system. Therefore, the use of rough set theory in combination with neural networks can reduce the size of the system and decrease the time of fault diagnosis, which will achieve more desirable results in fault diagnosis.

As a result, this paper processes an ELM network model for comprehensive inverter fault diagnosis using a knowledge-reduction method based on rough set (RS) theory. This paper’s main contributions are as follows: (1) Each internal IGBT operational state is determined by examining the power value changes associated with the positive and negative half-waves of each phase current of the three-level inverter. (2) A genetic algorithm (GA)-based binary granular matrix knowledge-reduction method is proposed. The decision attributes of the problem are derived through knowledge reduction under the assumption that the classification ability of the information system remains unchanged. (3) Create a fault detection model that combines GA-GrC and ELM neural networks, replace all attributes with reduction results, improve the classification performance of the ELM neural network, and thus improve the diagnosis speed, accuracy, and real-time performance of the detection system.

## 2. T-3L Inverter OC Fault Analysis

### 2.1. PWM Modulation and Operation Mode

**Figure 1:**
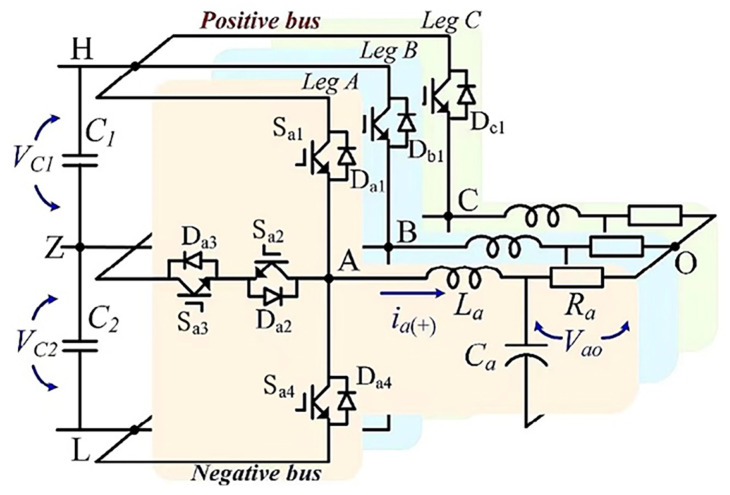
T-3L inverter topology.

Figure 1 depicts the circuit topology of a T-3L inverter. The *A*-phase bridge arm is used as an example, with four IGBTs and four freewheel diodes in reverse parallel with the IGBT to provide a current reverse conduction loop. The output voltage of the inverter is routed to the load via the LC filter. The three-phase bridge arm of the inverter contains 12 IGBTs, the switching state of which is controlled by the gate signal. When the gate signal is 1, the IGBT is turned on. When the gate signal is set to 0, the IGBT is turned off.

The T-3L inverter has three states when it is operating normally, as shown in Table 1. In this paper, pulse width modulation (PWM) is used to control the inverter switching state. Command signal 1 signifies switch-on, while 0 indicates switch-off. Activating switches $S_{a1}$ and $S_{a2}$ creates switching state *P*, resulting in a corresponding pole voltage $V_{AO}=+V_{dc}/2$. Switching state *O* is formed by turning on either $S_{a2}$ or $S_{a3}$, depending on the current direction, with the corresponding pole voltage $V_{AO}=0$. Activation of switches $S_{a3}$ and $S_{a4}$ produces switching state *N*, with a corresponding pole voltage $V_{AO}=-V_{dc}/2$. When the T-type inverter is operational, $V_{C_{1}}$ and $V_{C_{2}}$ oscillate at a low frequency in $V_{dc}/2$, with an oscillation period of 1/3 current cycle.

### 2.2. T-3L Inverter OC Fault Characteristics

**Figure 2:**
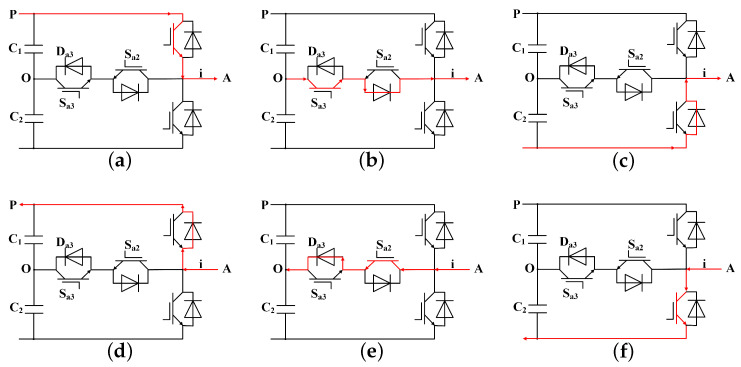
Switching state circuits based on the current direction for phase *A*: (**a**) Switching states *P* for i>0. (**b**) Switching states *O* for i>0. (**c**) Switching states *N* for i>0. (**d**) Switching states *P* for i<0. (**e**) Switching states *O* for i<0. (**f**) Switching states *N* for i<0.

To elucidate the operation of various fault diagnostic approaches, this section discusses the operation of a single-phase three-level T-type inverter topology. It is assumed that the current flowing from any one of the DC-link terminals to the inverter pole is considered a positive direction of the current. The red arrows in Figure 2 depict the current paths for different switching states in the positive and negative current scenarios.

Due to the symmetry of the circuit, only phase *A* is analyzed. The current direction from the inverter to the grid is considered positive (i>0). The figures below depict the current circuit of each switching device in the T-3L inverter in the event of an OC fault. The solid line represents the actual current flow path, while the dashed line illustrates the current flow path of $S_{a1}$ assuming normal conduction.

#### 2.2.1. An OC Fault Occurs on a Single IGBT

**Figure 3:**
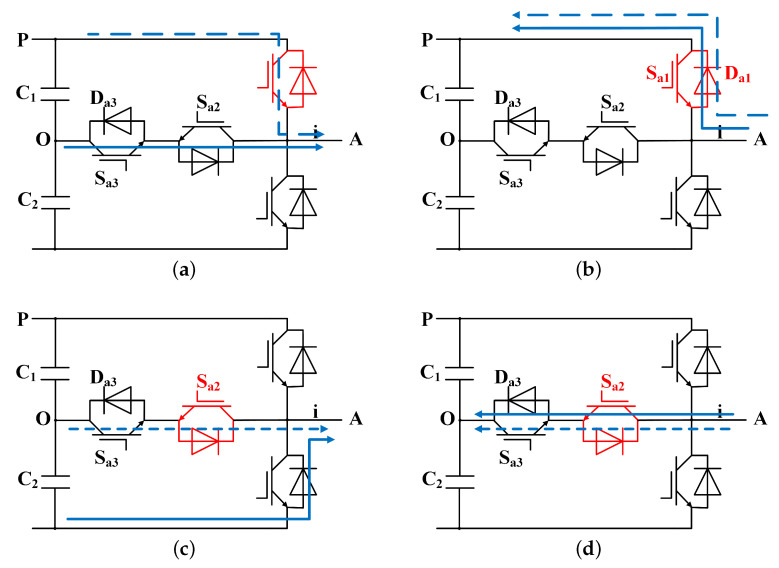
Single IGBT fault: (**a**) $I_{a}>0$, $S_{a1}$ fails. (**b**) $I_{a}<0$, $S_{a1}$ fails. (**c**) $I_{a}>0$, $S_{a2}$ fails. (**d**) $I_{a}<0$, $S_{a2}$ fails.

As shown in Figure 3a, when $I_{a}>0$, the open circuit of $S_{a1}$ causes the *A*-phase output state of the inverter change from *P* to *O*, and the discharge capacitance to change from $C_{1}$ to $C_{2}$, establishing $V_{C1}>V_{C2}$. At this point, the current can only be output after passing through $S_{a2}$ and $S_{a3}$ to the neutral point, where it quickly attenuates to zero, and the output power approaches 0*W*. When $I_{a}<0$, Figure 3b shows that the *A*-phase output state of the inverter will not change due to the open circuit of $S_{a1}$. The *A*-phase output current of the inverter will flow out from the negative terminal via the reverse shunt diode of the IGBT on the lower side, creating a charging situation to the direct current (DC) side. At this point, the power associated with the positive half-wave current will be negative.

When $S_{a2}$ fails, as shown in Figure 3c, and $I_{a}>0$, the open-circuit $S_{a2}$ has no effect on the output state of phase. When the current is negative, it is routed back to the DC side power supply via the inverse shunt diode on $S_{a4}$. At this point, only $C_{2}$ will discharge, and the output state will change from *O* to *P*, resulting in a halving of the output power amplitude.

#### 2.2.2. An OC Fault Occurs on Two IGBTs in the Same Phase

**Figure 4:**
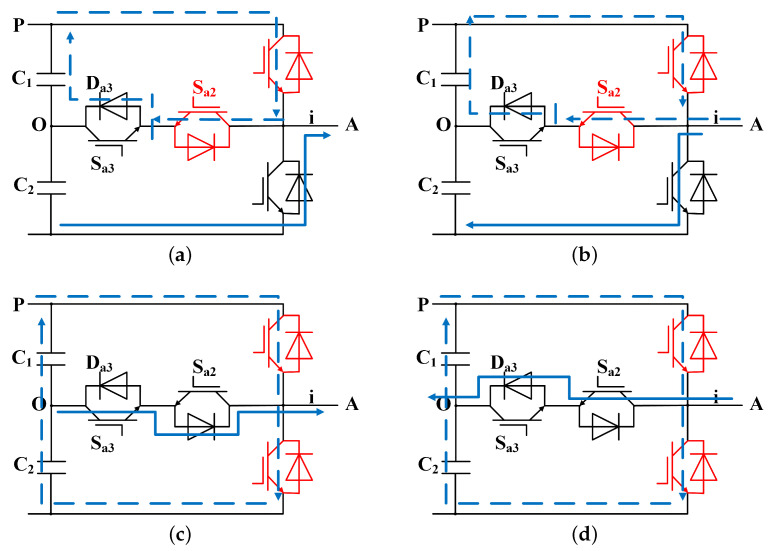
Two IGBTs fault in the same phase: (**a**) $I_{a}>0$, $S_{a1}$ and $S_{a2}$ fails. (**b**) $I_{a}<0$, $S_{a1}$ and $S_{a2}$ fails. (**c**) $I_{a}>0$, $S_{a1}$ and $S_{a4}$ fails. (**d**) $S_{a1}$ and $S_{a4}$ fails.

Consider the open circuit $S_{a1}$ and $S_{a2}$ as an illustration. When the load current direction is positive, as in Figure 4a, the current enters the negative terminal *N* of the DC bus and exits through the switch tube $S_{a4}$. The voltage between the two points is currently $-V_{dc}/2$, and the output terminal is connected to the negative terminal *N* of the DC bus. According to Figure 4b, When the load current direction is negative, the current enters the output end *A* of the inverter, passes through the inverse shunt diode of $S_{a4}$, and then enters the negative polar end *N* of the DC bus. The *A*-phase current only has the negative half-wave part, and the voltage is $-V_{dc}/2$. At the moment of the fault, the power of this phase dropped suddenly, accompanied by a significant increase in harmonics in the *B* and *C* phase currents, leading to varying degrees of power decline.

Experiments reveal that changes in current and power differ significantly from the fault characteristics mentioned above when two internal switches or external switches fail simultaneously. As an illustration, consider the OC fault between $S_{a1}$ and $S_{a4}$. According to Figure 4c,d, when the load current is flowing in the opposite direction, the current input end is different and exits through the reverse parallel diode of $S_{a2}$ and $S_{a3}$, respectively. As a result, the output end *A* is connected to the midpoint *O* of the DC bus, the voltage is 0*V*, and the power of this phase is 0*W* correspondingly.

Similarly, when the two internal switches $S_{a2}$ and $S_{a3}$ re disconnected, the gate-emitter bias voltage of $S_{a2}$ and $S_{a3}$ does not cause large oscillations of $V_{ce}$, resulting in reverse recovery characteristics similar to diodes for $S_{a1}$ and $S_{a4}$. The reverse recovery current and voltage peak fall and the loss is extremely low. Therefore, the *A*-phase power drops to 0*W* in an instant and then returns to a stable state. As a result, the magnitude of power is only second to the *B* and *C* phases.

#### 2.2.3. An OC Fault Occurs on Two IGBTs in the Different Phase

If $S_{a1}$ and $S_{b1}$ were to be disconnected, for instance, the current could only flow from the middle point *O* to the output end of phase *A* via the anti-parallel diodes of $S_{a2}$ and $S_{a3}$. The open circuit of $S_{a1}$ and $S_{b1}$ also affects phase *B* and phase *C* current, preventing the current from reaching $S_{c1}$ from the *P* end. As a result, the output power is 0*W*, and the current quickly decays to 0*A*. When the current direction is negative, the current flows into the output end, and $S_{c1}$ is normally switched on, whereas the output state of the other two places does not change due to the open circuit, allowing only negative half-wave current can occur and the output amplitude decreases. Therefore, the output power only fluctuates around 0*W*.

From the above fault characterization, it is clear that a fault in the power device will force a change in the current path of the faulty phase in the inverter, resulting in a change in its operating state, and this transfer between operating states can be represented and tracked using a finite state machine. Define the current direction and the power switch fault as state transfer rules, which are represented by logical variables $\delta_{\left(+/-\right)}$ and $F_{SXk}$, respectively. When the current direction is positive, $\delta_{\left(+\right)}=1$, and vice versa, $\delta_{\left(-\right)}=1$. $F_{Sa1}=1$ means $S_{a1}$ has an open circuit fault, $F_{Sa1}=0$ which means $S_{a1}$ is working normally. Taking phase *A* of the inverter as an example, the transfer of the circuit operation status in each operating mode is concluded in Table 2.

Based on the analysis above, the characteristics of single and double-tube faults can be used to diagnose other fault types and locate the fault phase and fault tubes.

## 3. Fault Characteristic Selection

The factors influencing the occurrence of faults are complex, and the fault sample data have many attributes and large dimensions, which leads to long data-processing time and makes fault classification difficult. Furthermore, there are a significant number of similar attributes in the fault data, and these similar attributes have an approximate influence on the fault classification results, with little difference. As a result, this paper proposes a method for reducing fault attributes, in which one main attribute replaces the approximate attribute for subsequent data classification.

### 3.1. Knowledge-Reduction Method Based on Granular Matrix

Professor Zadeh developed the concept of granular computing (GrC) [28]. Granular computing is a new computing paradigm that addresses difficult challenges. It uses organized thinking, structured problem-solving methodologies, and structured information processing models as research subjects. The primary idea is to use hierarchical degrees of granularity to abstract and refine complicated problems, resulting in many simpler problems to solve. Three theoretical models are highlighted: rough set, quotient space, and computing words. RS theory can analyze and express fuzzy knowledge, as well as extract hidden rules from large amounts of data for analysis and solution. Additionally, RS theory and other machine-learning algorithms are very complementary, and their combined advantages can be very beneficial.

The research object in the framework of RS theory is an information system composed of an object set and an attribute set. The information system is defined as *T*, i.e., T=(U,M∪N,V,f). where *U* is the collection of objects, also known as the universe, and *M* and *N* are sets of conditional and decision attributes, respectively. *V* and *f* represent range and information function collections, respectively. *A* set of knowledge includes all subsets. This “attribute-value” relationship results in a collection of decision tables. When redundant or unimportant knowledge is removed from the decision information system, the information system is said to be simplified.

Granulation and granular computing are the most fundamental problems in granular computing. Granulation is the division of a problem space into several subspaces or the classification of individuals in the problem space based on useful information and knowledge. Granules are the name given to these classes. The key to granular computing is to understand how to build a reasonable granular world and solve practical problems. However, representing the concept of rough sets with binary particles is a convenient and feasible algorithm model.

Let K=(U,R) represent a data-set and p∈R represent an equivalence relation on *U*, denoted as IND(R), where $U/P=\left\{z_{1},z_{2},\cdots ,z_{n}\right\}$. The granularity of *P* is denoted as GD(P), and its specific calculation formula is:

(1) $$GD\left(P\right)=\frac{|P|}{|U\times U|}=\sum_{i=1}^{n}\frac{|z_{i}{|}^{2}}{|U{|}^{2}}$$

The particle size of *P* represents its resolution. For ∀u,v∈U, when $\left(u,v\right)\in P$, *u* and *v* are indistinguishable under *P*, If it is indistinguishable, it belongs to a different p− equivalence class. It can be concluded that GD(P) represents the probability of p− indiscernibility of two randomly selected objects in *U*, and the higher the value, the lower the resolution ability. Define the resolution Dis(P) of knowledge *P* as:

(2) Dis(P)=1−GD(P)

Because of the diversity of each piece of knowledge and the complexity of its contents, this paper uses binary particles to represent each piece of knowledge. Let $U=\left\{u_{1},u_{2},\cdots ,u_{n}\right\}$ be the universe and *R* be the equivalence relation. Each equivalence class in U/R can be expressed by an *n* -bit binary string. If the ith bit is 0, $u_{i}$ does not belong to this granule; if it is 1, it means $u_{i}$ belongs to this granule.

### 3.2. Characteristic Selection

Based on the preceding understanding of the essential ideas of granular computing, this part describes the relevant operations of granules and develops the operational basis of the binary granular matrix knowledge-reduction methodology based on genetic algorithms.

When

(3) $$\begin{array}{cc} \; & U/\mathrm{IND}\left(M\right)=Y=\left\{Y_{1},Y_{2},\cdots ,Y_{i},\cdots ,Y_{m}\right\} \end{array}$$

(4) $$\begin{array}{cc} \; & U/\mathrm{IND}\left(N\right)=X=\left\{X_{1},X_{2},\cdots ,X_{i},\cdots ,X_{n}\right\} \end{array}$$

The binary particle matrix is defined as $\left\{X_{n\times t},Y_{m\times t},C_{n\times m}\right\}$, where $C_{n\times m}$ is the relation matrix of the attribute set *M* and *N*. That is

(5) $$\begin{array}{cc} \; & X_{n\times t}=\left(\begin{array}{c} X_{1}\\ \ldots \\ X_{n} \end{array}\right)=\left(\begin{array}{ccc} b_{11} & \ldots & b_{1t}\\ \ldots & \ldots & \ldots \\ b_{n1} & \ldots & b_{nt} \end{array}\right) \end{array}$$

(6) $$\begin{array}{cc} \; & Y_{m\times t}=\left(\begin{array}{c} Y_{1}\\ \ldots \\ Y_{m} \end{array}\right)=\left(\begin{array}{ccc} a_{11} & \ldots & a_{1t}\\ \ldots & \ldots & \ldots \\ a_{m1} & \ldots & a_{mt} \end{array}\right) \end{array}$$

(7) $$\begin{array}{cc} \; & C_{n\times m}=X\times Y \end{array}$$

Among them, $c_{ij}=\sum_{k=1}^{t}b_{ik}a_{kj},\left(i=1,2,\cdots ,n;j=1,2,\cdots ,m\right)$. *a* and *b* represent the binary strings under the corresponding set.

The relation matrix $C_{n\times m}$, whose value corresponds to the proportion of $Y_{i}$ elements in $X_{j}$, expresses a subordinate relation between all equivalent classes $X_{j}$ and $Y_{i}$. In this manner, the chromosome of the genetic algorithm can be sequentially mapped to each attribute. Each binary string represents a chromosome, and each binary particle, which has a value range of [0,1], represents a gene.

The dependence of the attribute β describes the compatibility of the decision information system, where

(8) $$\beta =\frac{\mathrm{card}\left(POS_{M}\left(N\right)\right)}{\mathrm{card}(U)}$$

In the formula, the number of elements in $POS_{M}\left(N\right)$ is represented by $\mathrm{card}\left({\mathrm{POS}}_{M}\left(N\right)\right)$, the number of elements in $POS_{M}\left(N\right)$ is known as the *M* positive domain of *N*, and the total number of elements in the universe *U* is represented by $card\left(U\right)$. When β=1, it is a compatible decision information system; otherwise, it is called an incompatible decision information system.

The set obtained after reduction is denoted as *Q*, and the set of irreducible relations contained in all reduced attribute sets is referred to as core attribute, i.e., the intersection of the reduced set $red\left(Q\right)$, denoted as $core\left(Q\right)$. The collection of significant attributes required for this knowledge is referred to as the core attribute. Verify the compatibility of other knowledge with kernel attributes based on the obtained core attributes and then compute the dependency to determine the minimal set of attributes. The specific algorithm is shown in Algorithm 1.
**Algorithm 1** Attribute Reduction Algorithm1:Input: Information System T=(U,M∪N,V,f);2:Calculate $core\left(Q\right)$: ∀p∈M, calculate β, all attributes with β<1 constitute $core\left(Q\right)$;3:RED(Q)=CORE(Q);4:Determine whether IND(RED(Q))=IND(Q) is valid. If so, proceed to step 7; otherwise, proceed to step 5;5:Calculate all values of x∈A−RED(A), recorded as $Sig_{RED(Q)}$, take $x_{1}$ to satisfy: $Sig_{RED(Q)}\left(x_{1}\right)=\max_{x\in Q-RED(Q)}\left\{Sig_{RED(Q)}\left(x\right)\right\}$;6:$RED\left(Q\right)=RED\left(Q\right)\cup \left\{x_{1}\right\}$, then proceed to step 4;7:Output minimum reduction RED(Q);

Finally, the fitness function is used to search. Assume that any object *U* has the conditional attribute *M*, and that its fitness is as follows [29].

(9) $$F\left(M\right)=\frac{n-l_{M}}{l}+\beta$$

where $l_{M}$ stands for the number of genes for which conditional attribute *M* has a value of 1, and β is the extent to which the decision attribute *N* is dependent on the attribute subset corresponding to conditional attribute *M*. It can be seen that the fitness function takes the number of subset elements and attribute dependency into account completely. Conditional attributes can be managed to evolve to the minimum attribute reduction set to achieve this.

For kernel attributes that cannot accurately describe all information, randomly select two attributes from set *U* for crossover, generating two new attributes. The new individual is formed by the intersection of attribute *p* and attribute *q* at gene *j*:

(10) $$\left\{\begin{array}{c} x_{pj}=x_{pj}\left(1-a\right)+x_{qj}a\\ x_{qj}=x_{qj}\left(1-a\right)+x_{pj}a \end{array}\right.$$

where *a* is a random number of [0, 1].

Randomly select attribute *X* from set *U*, mutate the gene *j* of that attribute, and the resulting new individual is:

(11) $$x_{kj}^{\prime }=\left\{\begin{array}{c} x_{kj}\times \left[1-c\times {\left(1-\frac{t}{t_{m}}\right)}^{2}\right],\;b\leq 0.5\\ x_{kj}\times \left[1+c\times {\left(1-\frac{t}{t_{m}}\right)}^{2}\right],\;b>0.5 \end{array}\right.$$

where *b* and *c* are random numbers between [0, 1], it is the current iteration number, and $t_{m}$ is the maximum number of iterations.

In each iteration, the best conditional attribute is preserved to prevent it from undergoing crossover and mutation again, ensuring the maximum inheritance of the conditional attribute. During each iteration, the worst attribute in the current population is replaced with the best attribute.

Continue the repetition until GA training reaches the maximum number of iterations, then determine the final kernel attribute set based on the fitness value.

**Figure 5:**
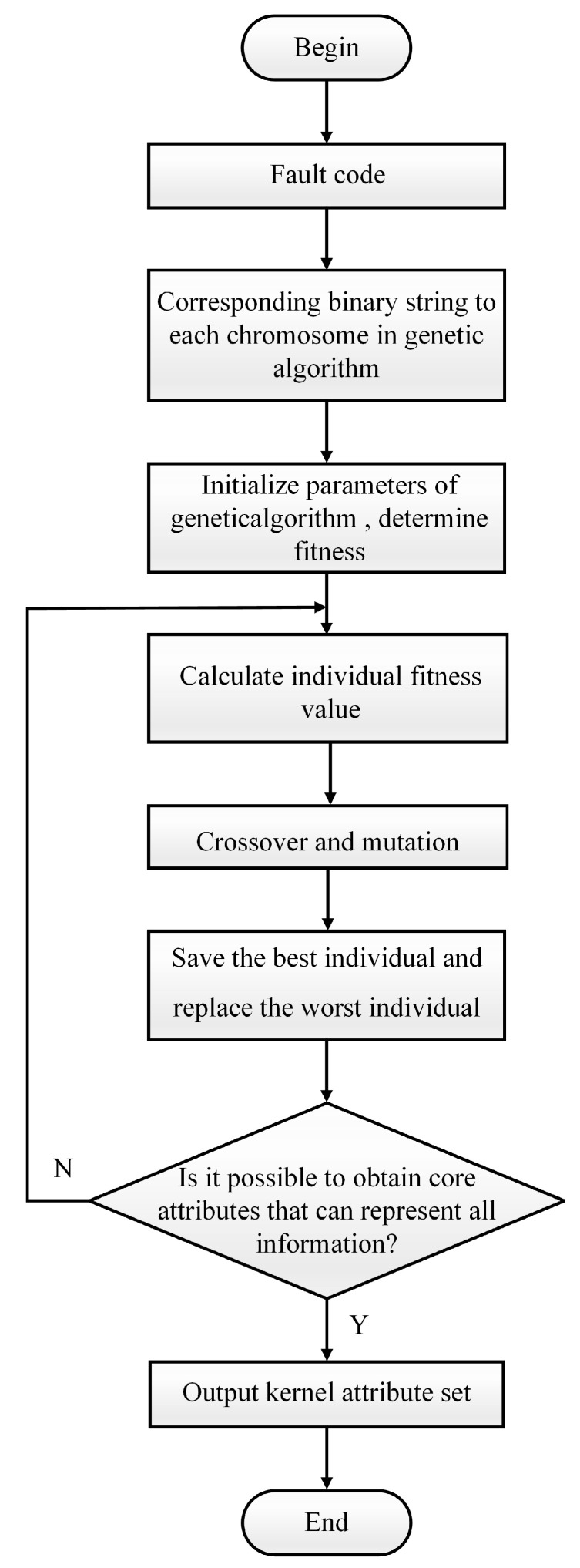
Flowchart of binary granular matrix knowledge-reduction algorithm based on genetic algorithm optimization.

Traditional genetic algorithms frequently use constant probabilities for crossover and mutation. As a result, the direct genetic operation of the traditional genetic algorithm on the population significantly slows convergence and fails to identify individual traits. To address this problem, this study adapts the probability values of crossover and mutation based on the population’s fitness value. This approach can improve the genetic evolutionary algorithm’s convergence speed and accuracy, as well as its global search capabilities while avoiding slipping into the local optimal solution. Figure 5 below depicts the flow chart for the binary granulation matrix knowledge-reduction approach based on genetic algorithm optimization.

### 3.3. Fault Detection Model Based on GA-GrC-ELM

The ELM neural network, also known as the feedforward neural network, uses the error between the output result and the real result to estimate the error of the previous layer of the output layer and then uses the error of this layer to estimate the error of the previous layer so that the error estimate of each layer is obtained repeatedly. The parameters of the ELM hidden layer can be set randomly, or a kernel function can be used as the hidden layer. ELM can only determine the output weight by computing the inverse of the parameter matrix *H*. The training process goes as follows:

(12) $$\sum_{i=1}^{n}T_{i}g\left(x_{j}\right)=\sum_{i=1}^{n}T_{i}g\left(V_{i}\cdot x_{j}+W_{i}\right)=O_{j}$$

In this formula, $V_{i}$ represents the weight from the input layer to the hidden layer, $W_{i}$ represents the system bias, $T_{i}$ represents the weight from the hidden layer to the output layer, *g* represents the activation function, *n* represents the size of the training set, $O_{j}$ represents the output value, i.e., the classification result. To infinitely approximate the real result of the training data, the classification result is consistent with the real result *P*, i.e., $\sum_{i=1}^{n}∥O_{j}-P_{j}∥=0$, so the formula can be obtained

(13) $$\sum_{i=1}^{n}T_{i}g\left(V_{i}\cdot x_{j}+W_{i}\right)=P_{j},\left(j=1,\cdots ,n\right)$$

Written in matrix form as HT=P

*H* is the output matrix of the hidden layer. The specific form is as follows:

(14) $$H\left(V_{1},\cdots ,V_{n},W_{1},\cdots ,W_{n},x_{1},\cdots ,x_{n}\right)={\left[\begin{array}{ccc} g(V_{1}\cdot x_{1}+W_{1}) & \cdots & g(V_{l}\cdot x_{1}+W_{l})\\ \cdots & \cdots & \cdots \\ g(V_{1}\cdot x_{n}+W_{1}) & \cdots & g(V_{l}\cdot x_{n}+W_{l}) \end{array}\right]}_{n\times l}$$

(15) $$T={\left[\begin{array}{c} T_{1}^{T}\\ \cdots \\ T_{l}^{T} \end{array}\right]}_{l\times m}$$

(16) $$P={\left[\begin{array}{c} p_{1}^{T}\\ \cdots \\ p_{l}^{T} \end{array}\right]}_{n\times m}$$

Among them, *n* represents the size of the training set, *l* represents the number of hidden layer nodes, g(x) represents the activation function, and g(x) requires wireless differentiability.

The goal of training the ELM model is to find the best *T* with the lowest training errors. The mathematical expression of the ELM model is as follows:

(17) $$\min{∥\varepsilon ∥}^{2}=s.t.\sum_{i=1}^{n}T_{i}g\left(V_{i}\cdot x_{j}+W_{i}\right)-P_{j}=\varepsilon_{j},j=1,\cdots ,n$$

Among them, $\varepsilon_{j}$ is the error between the category to which the jth sample belongs and the category determined by the model. By optimizing the parameters of the model, ${∥\varepsilon_{j}∥}^{2}$ obtains the minimum value.

**Figure 6:**
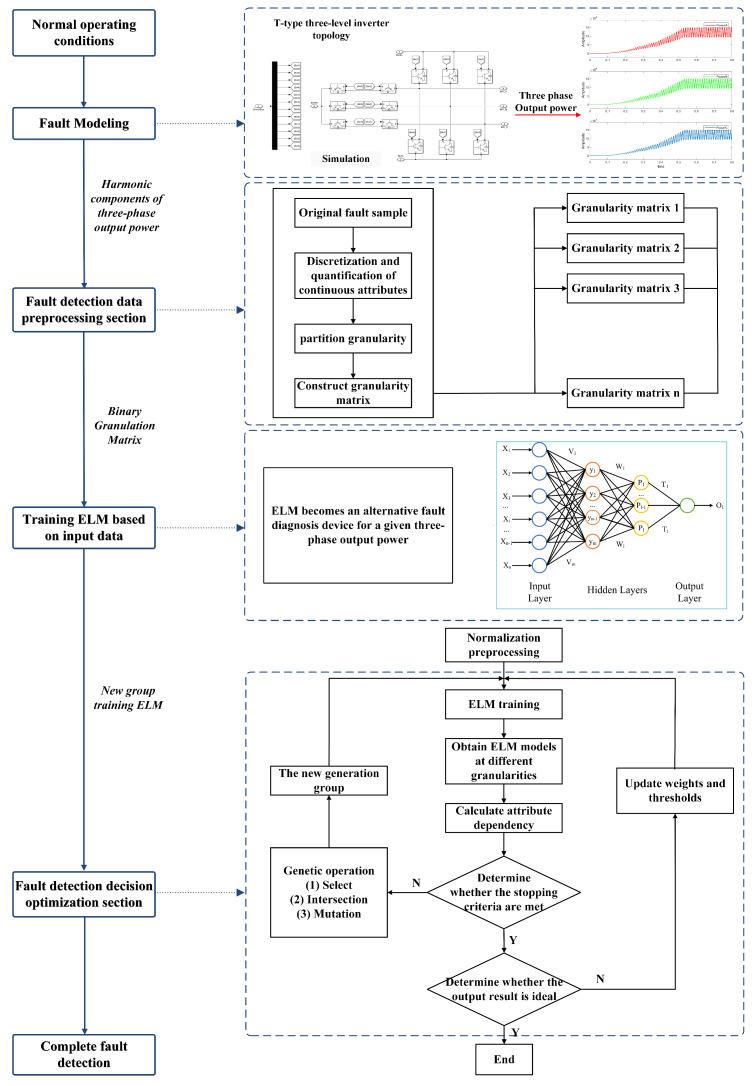
The proposed model structure.

Figure 6 depicts the proposed GA-GrC-ELM-based T-3L inverter fault-diagnosis structure diagram. The model primarily consists of a GA-GrC attribute reduction component and a neural network diagnosis component. First, using granular computing as the front-end information processor of the neural network, GrC can use its strong attribute reduction capabilities to eliminate duplication and create the smallest possible attribute set. To obtain the decision table for attribute reduction, the fault data of the T-3L inverter is then discretized and quantized using the clustering discretization method, and the repeated data are removed. The final step is to obtain the minimum attribute set for the final input fault sample using the grain matrix knowledge-reduction method, which is based on a genetic algorithm, adaptively changes the probability values of crossover and mutation according to the fitness value of the population to enhance its global search capability. Second, a GA-GrC-ELM neural network model needs to be constructed. The essential thing is to establish the hidden layer parameters for two neural networks to train the GA-GrC-ELM neural network using the decision attributes as the output and conditional attributes from the reduced training fault data as the input. Based on the minimum attribute set, the test sample verifies the GA-GrC-ELM network.

## 4. Experiment and Simulation

**Figure 7:**
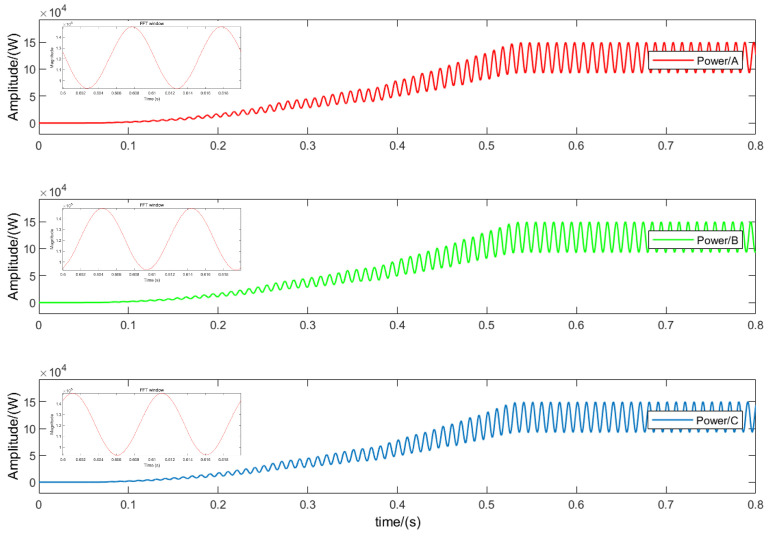
MATLAB simulation results under normal operation.

To validate the accuracy and real-time performance of the T-type three-level inverter fault-diagnosis solution based on output power, an offline simulation model was constructed using MATLAB/Simulink. The simulation parameters of the system are shown in Table 3. Figure 7 illustrates the output power of the T-type three-level inverter during normal operation. Due to the periodic averaging method, the output power undergoes a transition before entering a new steady-state process.

**Figure 8:**
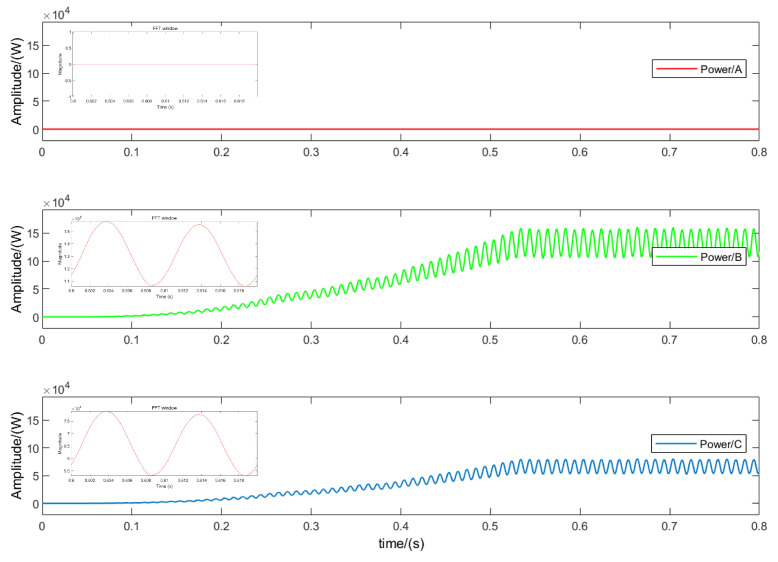
MATLAB simulation results under the condition of an open-circuit fault in $S_{a1}$.

Figure 8 shows the simulation results of the OC fault of $S_{a1}$ in phase *A*. When the output power enters a new steady-state process, it can be seen that compared with the normal state, after the OC fault of $S_{a1}$ occurs, the output power is 0, and at the same time, other phases are accompanied by harmonics. This is because normally, the *A* phase works in the *P* state; the forward current flows through the DC bus and is transmitted to the load through the bridge arm switch, and the bridge arm outputs a positive level. However, when an open-circuit fault occurs in $S_{a1}$, the *A* phase bridge arm output cannot Connected to the DC bus, and the forward current will freewheel through the midpoint switch $S_{a3}$ and the diode $D_{a2}$. At this time, the *A* phase cannot work in the *P* state, and the *P* state is replaced by the *O* state.

**Figure 9:**
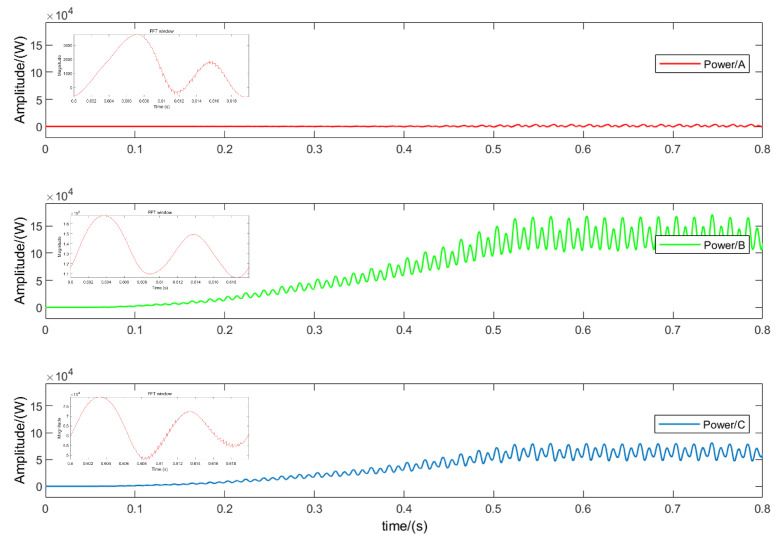
MATLAB simulation results under the condition of an open-circuit fault in $S_{a1}$ and $S_{a2}$.

Figure 9 shows the simulation results of the OC fault of phase *A*$S_{a1}$ and $S_{a2}$. Since the forward current can only continue to flow through the lower arm diode $D_{a4}$ after the fault, the operating state changes from *P* to *N*. It can be seen that when multiple IGBTs have OC faults at the same time, the power change is obviously different from the fault characteristics of a single IGBT OC fault. The simulation results are consistent with the theoretical analysis.

### 4.1. Dataset Selection

Since analyzing the power waveform and obtaining the fault characteristic signal in the time domain requires much calculation, the MATLAB library function FFT is used for the spectrum analysis of the three-phase output power waveform, and the amplitude and phase angle of each harmonic wave are obtained, with 50 Hz as the base frequency. By integrating and combining simulation results, it is discovered that the DC component, fundamental harmonic, and second harmonic of the three-phase power signal contain most of the information about various faults. As a result, the DC component, fundamental amplitude $A_{1}$, fundamental phase angle $\varphi_{1}$, second harmonic amplitude $A_{2}$, and second harmonic phase angle $\varphi_{2}$ are chosen as the input characteristic signals of the neural network.

#### 4.1.1. Data Discretization Processing

The experimental part of this paper randomly selects 330 sets of fault-type data and divides the training set, validation set, and test set according to the ratio of 8:1:1. The final training set has 264 sets of fault-type data, and the validation set and test set contain 33 sets of fault-type data, respectively. Since the particle computation attribute reduction is based on discrete data, 30 sets of training sample data are randomly selected for discretization using the cluster discretization method in this paper. The decision table is obtained by further quantifying the discretized data and removing duplicate samples, as listed in Table 4.

#### 4.1.2. Data Reduction

In Table 4, *U* is the universe, $M=\left\{A_{DC},A_{A_{1}},A_{\varphi_{1}},\cdots ,B_{A_{2}},B_{\varphi_{2}},\cdots C_{A_{2}},C_{\varphi_{2}}\right\}$ is the conditional attributes, and N={1,2,3,4} is the decision attributes, respectively, represents the number of faulty devices.

*Y* is the identity matrix, which can be obtained from Equations (5)–(8):

(18) $$X_{4\times 30}=\left(\begin{array}{c} X_{1}\\ X_{2}\\ X_{3}\\ X_{4} \end{array}\right)=\left(\begin{array}{c} 111110000000000000000000000000\\ 000001111111100000000000000000\\ 000000000000011111111000000000\\ 000000000000000000000111111111 \end{array}\right)$$

(19) $$C=Y\times X^{\prime }=\left(\begin{array}{c} 111110000000000000000000000000\\ 000001111111100000000000000000\\ 000000000000011111111000000000\\ 000000000000000000000111111111 \end{array}\right)$$

(20) $$\mathrm{card}\left({\mathrm{POS}}_{M}\left(N\right)\right)=\sum_{NE(i)=1}c_{ij}=30$$

However, card(U)=30, i.e., β=1, which means that decision Table 4 is a compatible decision table.

First, by removing a single attribute, such as attribute $A_{DC}$, we can obtain:

(21) $$C=Y_{M-A_{DC}}\times X^{\prime }=\left(\begin{array}{c} 11210000000000000000000000\\ 00000111111100000000000000\\ 00001010000011111000000000\\ 00000000000000000011112111 \end{array}\right)$$

(22) $$\mathrm{card}\left({\mathrm{POS}}_{M-A_{DC}}\left(N\right)\right)=\sum_{NE(i)=1}c_{ij}=26$$

(23) $$\beta_{IND}\left(A_{A_{1}},\ldots ,A_{\varphi 2},B_{DC},B_{A_{1}},\ldots ,B_{\varphi 2},C_{DC},C_{A_{1}},\ldots ,C_{\varphi 2}\right)=\frac{\mathrm{card}\left({\mathrm{POS}}_{M-A_{DC}}\left(N\right)\right)}{\mathrm{card}(U)}=\frac{26}{30}<1$$

It indicates that the attribute $A_{DC}$ depends on *N* and is irreducible.

Similarly, other attributes were removed one at a time to test whether the attribute and its related attribute set could represent complete information based on attribute dependency.

**Figure 10:**
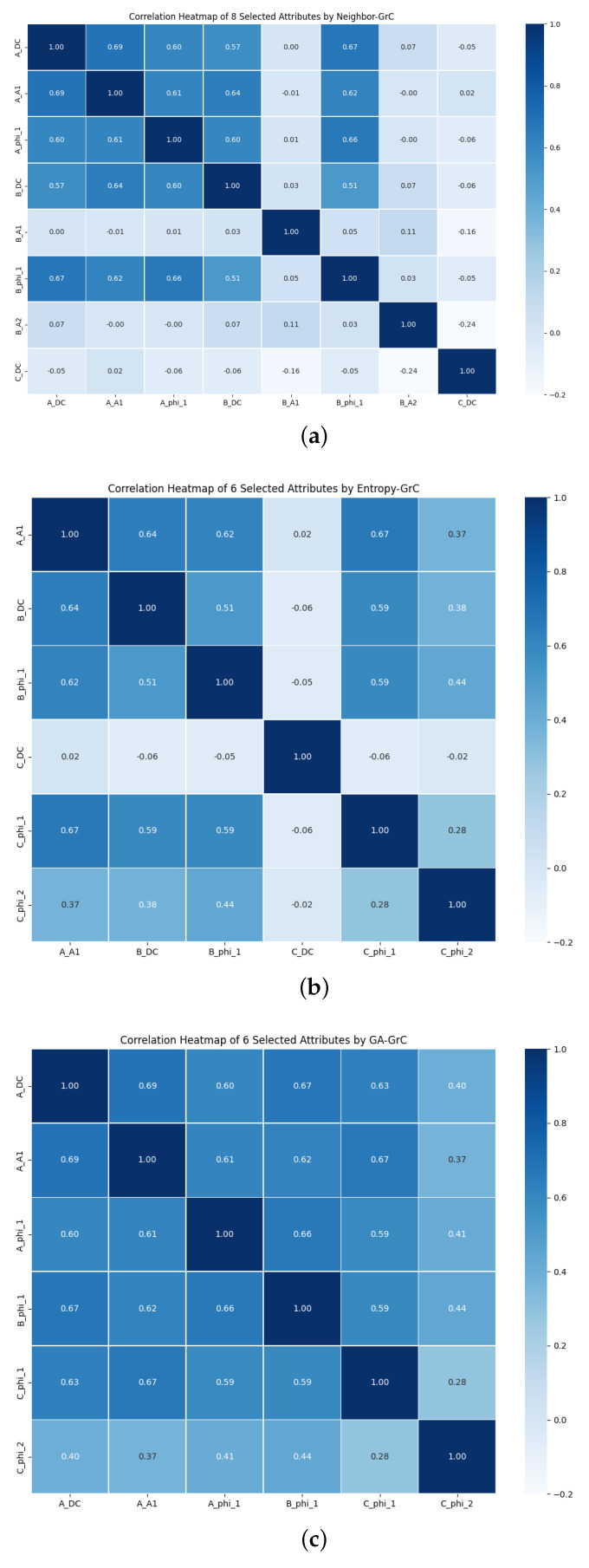
The capability to validate attribute reduction for three algorithms. (**a**) Neighbor−GrC, (**b**) Entropy−GrC, (**c**) GA−GrC.

This article is grounded in the concept of binary granular representation of rough sets, utilizing mutual information attribute reduction algorithms based on Neighbor rough sets attribute reduction algorithm, entropy-based rough set attribute reduction algorithm, and a proposed genetic algorithm (GA)-based rough set attribute reduction algorithm. The comparative experiment of attribute reduction results is presented in Table 5. Additionally, Figure 10 illustrates the testing capabilities of the three algorithms in assessing the attribute reduction performance of rough sets under various discrimination conditions. It is evident that the GA algorithm significantly reduces the number of optimal attribute sets on this dataset compared to the other two algorithms, demonstrating the stronger attribute reduction ability of the GA-GrC algorithm.

### 4.2. Result Analysis

**Figure 11:**
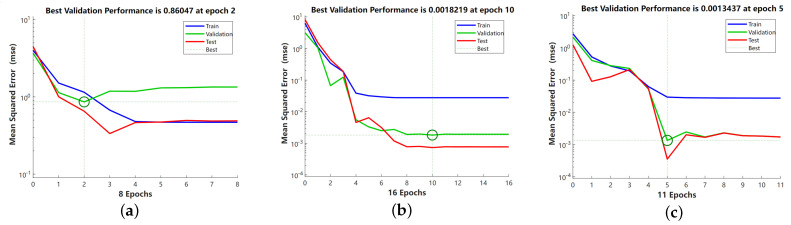
Sub-classifier training error curve. (**a**) Neighbor−GrC−ELM, (**b**) Entropy−GrC−ELM, (**c**) GA−GrC−ELM.

Figure 11 shows the training error curves of the binary grain matrix attribute approximation performance model for different optimization conditions on the training set, validation set, and test set, where the system automatically selects the stopping moment for the number of iterations in the adaptive condition. Where Train is the mean square error on the training set, Validation is the mean square error on the validation set, and Test is the mean square error on the test set. With the increase of epoch, the mean square error gradually stabilizes and oscillates in a small range, Although the ELM neural network based on Neighbor-GrC optimization converges quickly, the accuracy is far from the actual results. The ELM neural network based on Entropy-GrC optimization reaches the minimum convergence accuracy in the 10th iteration process. The ELM based on GA-GrC optimization proposed in this article has a minimum convergence after 5 iterations. Obviously, the GA-GrC-ELM neural network has a faster convergence speed and smaller convergence error because it introduces the crossover and mutation operators of GA during training, which expands the search space of the algorithm. The complementary advantages of genetic algorithm and binary granulation matrix knowledge reduction are improving the search ability and convergence speed, avoiding falling into local optimum, and improving the accuracy of the optimal solution.

**Figure 12:**
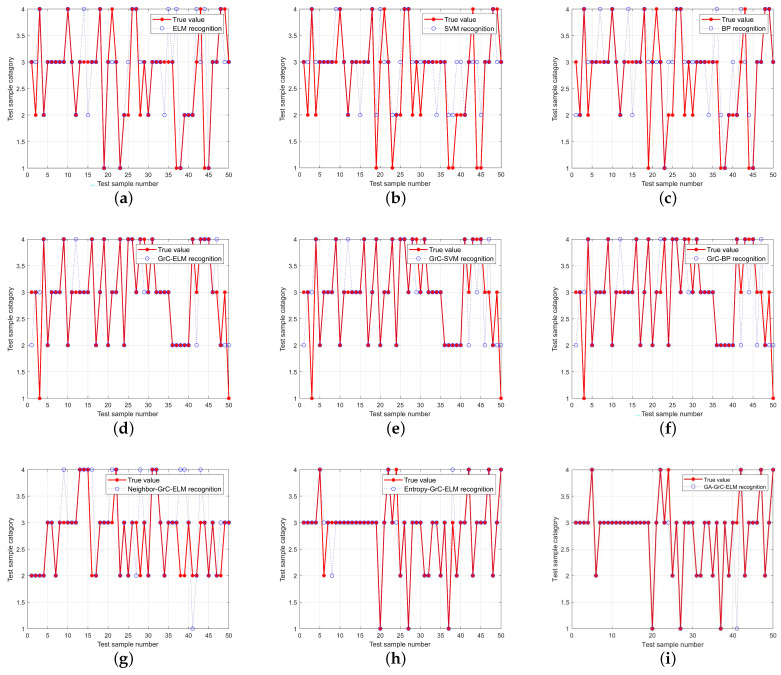
The classification results of data-set outcomes for each optimization method. (**a**) ELM, (**b**) SVM, (**c**) BP, (**d**) GrC−ELM, (**e**) GrC−SVM, (**f**) GrC−BP, (**g**) Neighbor−GrC−ELM, (**h**) Entropy−GrC−ELM, (**i**) GA−GrC−ELM.

To verify the performance of the genetic algorithm-based binary grain matrix approximation proposed in this paper, the data sets before and after the reduction were used as the input data of the ELM network, respectively, and the network was trained with the maximum training times set to 1000, the learning rate set to 0.01, and the minimum error of the training target set to 0.01%. In Figure 12, Figure 12a–c are the fault-diagnosis results of the original dataset based on different neural networks. In comparison, since the input weights of ELM are random and fixed, there is no need for an iterative solution. It is necessary to solve the weights from the hidden layer to the output layer. Therefore, compared with the BP algorithm and SVM algorithm, the ELM neural network has a smaller training step within the specified range and a higher accuracy. After rough set reduction based on granular computing, as shown in Figure 12d–f in the figure, the accuracy of each neural network has been significantly improved. It can be seen that the front-end processing of granular computing can effectively achieve data reduction, reducing redundant information and significantly improving the training accuracy of the neural network. Demonstrates the effectiveness of our proposed GrC algorithm. Figure 12g–i show the classification performance of binary granulation matrices based on different optimization conditions. The results prove that the binary granulation matrix reduction method based on a genetic algorithm expands the search space and avoids falling into local optimality. This paper proposes that the binary granulation matrix reduction performance based on a genetic algorithm is the best.

Table 6 compares the performance of different algorithms in terms of accuracy, mean square error, and running time. In terms of accuracy, the GA-GrC-ELM algorithm proposed in this paper achieves 98%, which is significantly better than BP, SVM, and ELM algorithms. Although the accuracy of other algorithms also improved after the addition of GrC, it was still inferior to the algorithm in this paper. In addition, the algorithm in this paper also performs well in terms of mean square error and running time. Considered together, the GA-GrC-ELM-based algorithm can diagnose the T-type three-level inverter faults faster and more effectively.

In summary, the T-type three-level inverter fault-diagnosis method based on GA-GrC-ELM proposed in this paper fully utilizes the ability of granular computing theory to remove redundant information and combines the genetic algorithm to automatically calculate the fault diagnosis based on the fitness value of the population. It adapts to changing the probability values of crossover and mutation, effectively enhances its global search capability, and effectively solves the problem of diagnostic accuracy caused by the complex training samples and high dimensions of neural networks.

## 5. Conclusions

In this paper, a GA-GrC-ELM-based fault-diagnosis method for T-3L inverters is presented. By measuring the power corresponding to the positive and negative half-waves of each phase current, the inverter OC fault is correctly identified and classified. The classification outcomes are then discretized and normalized to create a decision table for an input neural network model. The fault-diagnosis decision-making system is reduced using the granular matrix knowledge-reduction algorithm, which takes into account the various influences of each granularity. In addition, the reduction performance is optimized through adaptive functions to delete redundant attributes. The results of the experiments demonstrate that the GA-GrC-ELM algorithm resolves issues with the conventional single neural network model, such as its slow running speed, extensive training dataset, and challenging convergence. It offers more advantages in terms of fault-diagnosis precision and can better simulate judgment. Since the field environment is complex and changeable, there are many unexpected causes for the occurrence of a certain fault, and at the same time, there is a coupling relationship between the faults. Future work can consider the quantitative and directional comprehensive analysis of the fault occurrence and evolution mechanism in inverters from the perspectives of mathematical derivation as well as actual operating conditions.

## Figures and Tables

**Table 1:** The connection between output level and switch-on/off.

Output State	Output Level	$S_{a1}$	$S_{a2}$	$S_{a3}$	$S_{a4}$
P	$V_{dc}/2$	1	1	0	0
N	0	0	1	1	0
O	$-V_{dc}/2$	0	0	1	1

**Table 2:** Summary of T-inverter *A*-phase operation status under normal and fault conditions.

Initial State	Transfer Rules	Current Flow through Nodes	Output Level	End State
Mode I:State [P]	$F_{Sa1}=F_{Sa2}=0$	O-P-$U_{a}$	0.5$V_{dc}$	[P]
	$F_{Sa1}=1$, $\delta_{\left(+\right)}=1$	O-$U_{a}$	0	[O]
	$F_{Sa2}=1$	O-P-$U_{a}$	0.5$V_{dc}$	[P]
	$F_{Sa1}=F_{Sa2}=1$, $\delta_{\left(+\right)}=1$	O-N-$U_{a}$	−0.5$V_{dc}$	[N]
Mode II:State [O]	$F_{\mathrm{Sa}2}=F_{\mathrm{Sa}3}=0$	O-$U_{a}$	O-$U_{a}$	[O]
	$F_{\mathrm{Sa}2}=1$, $\delta_{\left(+\right)}=1$	O-N-$U_{a}$	−0.5$V_{dc}$	[N]
	$F_{Sa3}=1$, $\delta_{\left(+\right)}=1$	O-$U_{a}$	0	[O]
Mode III:State [N]	$F_{\mathrm{Sa}3}=F_{\mathrm{Sa}4}=0$	$U_{a}$-N-O	−0.5$V_{dc}$	[N]
	$F_{Sa4}=1$, $\delta_{\left(-\right)}=1$	$U_{a}$-O	0	[O]
	$F_{Sa3}=1$	$U_{a}$-N-O	−0.5$V_{dc}$	[N]
	$F_{\mathrm{Sa}3}=F_{\mathrm{Sa}4}=1$, $\delta_{\left(-\right)}=1$	$U_{a}$-P-O	0.5$V_{dc}$	[P]
Mode IV:State [O]	$F_{\mathrm{Sa}2}=F_{\mathrm{Sa}3}=0$	$U_{a}$-O	0	[O]
	$F_{Sa3}=1$, $\delta_{\left(-\right)}=1$	$U_{a}$-P-O	0.5$V_{dc}$	[P]
	$F_{Sa2}=1$, $\delta_{\left(-\right)}=1$	$U_{a}$-O	0	[O]

**Table 3:** Parameters of simulation model.

Parameters	Value	Unit
Grid line voltage	380	V
Power frequency	50	Hz
load resistance	0.2	Ω
DC side capacitance	10,000	uF
AC side inductance	0.1	mH
AC side resistance	0.1	Ω
DC side voltage	400	V

**Table 4:** The discretized decision table.

*U*	$A_{\mathrm{DC}}$	$A_{A_{1}}$	$A_{\varphi_{1}}$	$A_{A_{2}}$	$A_{\varphi_{2}}$	$B_{\mathrm{DC}}$	$B_{A_{1}}$	$B_{\varphi_{1}}$	$B_{A_{2}}$	$B_{\varphi_{2}}$	$C_{\mathrm{DC}}$	$C_{A_{1}}$	$C_{\varphi_{1}}$	$C_{A_{2}}$	$C_{\varphi_{2}}$	*N*
1	3	2	2	1	1	4	2	2	2	2	3	2	2	2	4	1
2	4	1	1	3	3	4	1	3	2	1	1	1	1	3	1	1
3	2	2	2	1	1	3	2	1	4	2	3	2	2	2	1	1
4	2	2	2	1	1	3	2	1	4	2	3	2	2	2	1	1
5	4	1	3	3	3	4	1	1	2	1	1	1	2	3	1	1
6	1	2	4	3	2	3	1	3	2	1	1	3	1	2	1	2
7	1	2	2	3	2	4	2	3	2	1	4	2	2	1	1	2
8	1	2	1	3	2	3	1	1	2	1	1	3	2	2	1	2
9	2	2	2	1	1	3	2	1	4	2	3	2	2	2	1	2
10	4	1	1	2	3	3	3	4	2	1	1	3	1	1	1	2
11	1	3	2	3	4	1	4	4	1	2	2	2	3	4	1	2
12	3	2	2	1	1	1	2	4	1	1	3	2	2	3	4	2
13	2	4	1	4	1	2	3	4	3	2	3	3	3	2	4	2
14	1	2	4	3	2	3	1	3	2	1	1	3	1	2	1	3
15	1	2	1	3	2	3	1	1	2	1	1	3	2	2	1	3
16	1	2	1	3	2	2	2	3	3	4	2	2	1	4	1	3
17	4	3	1	2	4	1	4	4	1	1	2	2	3	4	1	3
18	4	1	1	2	1	1	3	3	4	1	4	3	1	1	4	3
19	2	1	1	4	1	2	3	4	3	2	1	4	3	2	4	3
20	4	1	1	2	3	1	1	4	3	2	1	4	1	3	4	3
21	2	2	2	4	1	1	2	3	1	2	1	2	2	3	4	3
22	1	2	3	3	2	2	3	2	3	1	4	1	4	1	1	4
23	1	2	1	3	2	2	1	2	3	1	4	1	4	1	1	4
24	1	2	4	3	2	2	1	2	3	1	4	3	4	1	1	4
25	1	2	3	3	2	2	2	4	3	3	2	4	4	4	4	4
26	2	3	1	1	3	2	2	1	3	3	1	4	3	2	2	4
27	2	3	1	1	3	2	2	1	3	3	1	4	3	2	2	4
28	2	4	1	4	3	2	2	1	3	4	2	4	1	4	2	4
29	1	3	1	2	3	2	2	4	3	3	2	4	1	1	2	4
30	2	4	3	4	4	2	3	1	3	2	2	2	1	4	3	4

**Table 5:** Performance evaluation of different rough set reduction techniques.

Algorithm	Number of Condition Attributes after Reduction	Condition Attribute after Reduction	Reduction Ratio (%)	Average Running Time (s)	Characteristic
Neighbor-GrC ^1^	8	$A_{DC},\;A_{A_{1}},\;A_{\varphi_{1}},\;B_{DC},\;$ $B_{A_{1}},\;B_{\varphi_{1}},\;B_{A_{2}},\;C_{DC}$	46.67	2.9873	Avoid the problem of not being able to determine the domain radius of the original neighborhood rough set.
Entropy-GrC ^2^	8	$A_{DC},\;A_{A_{1}},\;A_{\varphi_{1}},\;B_{DC},\;$ $B_{\varphi_{1}},\;C_{DC},\;C_{\varphi_{1}},\;C_{\varphi_{2}}$	46.67	5.3436	High time complexity when there are many condition attributes.
GA-GrC ^3^	6	$A_{DC},\;A_{A_{1}},\;A_{\varphi_{1}},\;B_{\varphi_{1}}$ $C_{\varphi_{1}},\;C_{\varphi_{2}}$	60.00	3.7221	Complexity of fitness function calculation.

^1^ Mutual Information Attribute Reduction Based on Neighbor Domain Rough Set. ^2^ Attribute reduction of rough set based on Entropy. ^3^ Attribute Reduction of Rough Set Based on Genetic Algorithm.

**Table 6:** Training results of different algorithm.

Algorithm	Train Accuracy	Test Accuracy	MSE	Time
BP	74.1551	54	0.42	0.377
SVM	80.6429	66	0.78	73.611
ELM	80.6897	66	0.58	0.473
GrC-BP	86.9565	72	0.3782	0.403
GrC-SVM	85.7143	70	0.48	0.41
GrC-ELM	85	80	0.36	0.345
Neighbor-GrC-ELM	99.1667	84	0.86	0.447
Entropy-GrC-ELM	87.5	90	0.18	0.993
GA-GrC-ELM	92.1769	98	0.13	0.17

## Data Availability

The data presented in this study are available on request from the corresponding author. The data are not publicly available due to potential commercial values.
